# Transfection of RNA from Organ Samples of Infected Animals Represents a Highly Sensitive Method for Virus Detection and Recovery of Classical Swine Fever Virus

**DOI:** 10.1371/journal.pone.0126806

**Published:** 2015-05-11

**Authors:** Denise Meyer, Stefanie Schmeiser, Alexander Postel, Paul Becher

**Affiliations:** EU and OIE Reference Laboratory for Classical Swine Fever, Institute of Virology,Department of Infectious Diseases, University of Veterinary Medicine Hannover,Hannover, Germany; The University of Texas Medical Branch, UNITED STATES

## Abstract

Translation and replication of positive stranded RNA viruses are directly initiated in the cellular cytoplasm after uncoating of the viral genome. Accordingly, infectious virus can be generated by transfection of RNA genomes into susceptible cells. In the present study, efficiency of conventional virus isolation after inoculation of cells with infectious sample material was compared to virus recovery after transfection of total RNA derived from organ samples of pigs infected with Classical swine fever virus (CSFV). Compared to the conventional method of virus isolation applied in three different porcine cell lines used in routine diagnosis of CSF, RNA transfection showed a similar efficiency for virus rescue. For two samples, recovery of infectious virus was only possible by RNA transfection, but not by the classical approach of virus isolation. Therefore, RNA transfection represents a valuable alternative to conventional virus isolation in particular when virus isolation is not possible, sample material is not suitable for virus isolation or when infectious material is not available. To estimate the potential risk of RNA prepared from sample material for infection of pigs, five domestic pigs were oronasally inoculated with RNA that was tested positive for virus rescue after RNA transfection. This exposure did not result in viral infection or clinical disease of the animals. In consequence, shipment of CSFV RNA can be regarded as a safe alternative to transportation of infectious virus and thereby facilitates the exchange of virus isolates among authorized laboratories with appropriate containment facilities.

## Introduction

Classical swine fever (CSF) is one of the most important diseases of pigs and wild boar worldwide. It is caused by Classical swine fever virus (CSFV), a pestivirus belonging to the family *Flaviviridae* [1]. CSF outbreaks are associated with enormous economic losses, which are a result of the disease itself as well as of implemented control measures including stamping-out strategy and trade restrictions in the European Union (EU) [2, 3]. To confirm a CSF outbreak in the EU, National Reference Laboratories and the EU Reference Laboratory (EURL) have to follow the guidelines as laid down in the Technical Annex of European Commission Decision 2002/106/EC (EU Diagnostic Manual for CSF) [4, 5]. Currently, virus isolation on susceptible cells as well as detection of viral antigen and virus genome are used for CSF diagnosis. Also in case of previous diagnosis of CSF, it is recommended to perform virus isolation. New CSFV isolates have to be sent to the EURL for detailed characterization and are archived along with available epidemiological and sequence information. The CSF virus collection and the corresponding CSF database became an important tool for supporting the common efforts to eradicate CSF in the EU by providing a platform to exchange isolate-related information and by supporting molecular characterization of new CSFV isolates [6–10]. Generally, virus isolation is labor and time intensive and therefore only applicable for the analysis of small sample numbers. Leucocytes, non-coagulated blood and organ samples (lymph node, kidney, spleen, and tonsil) are preferred sample material [4, 5]. However, virus isolation from organ samples is often challenging due to toxic effects on cell culture. Furthermore, microbial contamination can occur after inoculation of cells with organ suspension despite high concentrations of antibiotics in cell culture medium. However, for detailed virus characterization including determination of virulence, isolation of infectious virus is indispensable.

Pestiviruses contain a single-stranded RNA genome of positive polarity. The genome encompasses one open reading frame which is translated into one polyprotein. Translation and replication of the virus are directly initiated in the cellular cytoplasm after uncoating of the viral genome [11, 12]. Apart from the classical way of infection, virus can be recovered after transfer of viral genomic RNA into cells by liposome-mediated transfection reagents or electroporation. Such transfection methods are the basis for reverse-genetic approaches which allow the generation of infectious virus from *in vitro* transcribed RNAs derived from full-length cDNA clones. Reverse-genetics has been successfully used for molecular characterization of CSFV and related pestiviruses including detailed analyses of genome organization, translation and biosynthesis of viral proteins as well as studies on RNA recombination, cytopathogenicity, and virulence [13–19]. In addition, RNA transfection has also been applied to rescue infectious foot-and-mouth disease virus (FMDV [20–23]) and CSFV [23] from sample material. However, a direct comparison of conventional virus isolation and RNA transfection for virus rescue using sample material of CSFV infected pigs has not been reported so far. In particular for sample material with high microbial contamination potential in cell culture (e.g. organ samples) RNA transfection might represent a good alternative to conventional virus isolation.

In view of the fact that shipment of samples from animals affected with list A diseases of the World Animal Health Organization (Office International des Epizooties; OIE) requires special biosafety precautions, it has been discussed to combine shipment of non-infectious sample material with recovery of infectious virus upon arrival of samples at authorized laboratories [21, 23]. In this context it has been reported that RNA prepared from infectious sample material using Trizol or phenol-chloroform preparation methods may represent a simplified and less expensive alternative to shipment of infectious sample material [23]. However, classification of RNA samples as biological non-hazardous material referred to studies on inoculation of susceptible cells with purified viral RNA. So far, it has not been experimentally examined whether inoculation of susceptible host species with RNA isolated from CSFV positive samples via the oronasal route can lead to infection and disease.

The major goals of the present study were to analyze if RNA transfection can be used as an additional diagnostic approach for isolation of infectious CSFV and whether RNA derived from infectious material poses a threat for pigs. For this purpose, the detection limits of virus rescue by RNA transfection and conventional virus isolation were compared to each other. Both, organ samples of experimentally infected pigs as well as field samples were tested. Furthermore, oronasal inoculation of pigs with RNA, prepared from whole blood of a highly viremic animal, was performed to determine whether accidental contact of pigs with CSFV RNA poses a threat with respect to biosecurity.

## Material and Methods

### Samples

Organ samples (spleen, kidney, tonsil, and lymph node) of four weaner pigs, which were experimentally infected with different CSFV isolates were analyzed. One animal was infected with cell culture supernatant of CSFV isolate “Rösrath” (CSF1045), two with cell culture supernatant of CSFV strain “Brescia” (CSF0947) and one with whole blood taken from an animal infected with CSFV strain “Koslov”, respectively. These samples were obtained from the organ sample collection of the EURL for CSF (Hannover, Germany). The respective animal experiment for obtaining the samples was notified by the Specialized Department of Animal Welfare Service of the Lower Saxony State Office for Consumer Protection and Food Safety (LAVES; http://www.state-office-for-consumer-protection-and-food-safety.niedersachsen.de/portal/live.php?navigation_id=26144&article_id=73766); Permit Number: LAVES AZ 08A 538) according to the German animal welfare act (Tierschutzgesetz, 25 Mai 1998, § 8a, Abs. 1 u. 2).

In addition, eight field samples [organ suspension, diluted 1:10 in phosphate buffered saline (PBS)] were tested, which were taken from pigs that were culled during a CSF outbreak in Serbia in November 2010 (sample no.: 154–1 to 154–8). Since these samples were collected in the context of CSF control measures no ethical approval was required. The samples were sent by the Scientific Institute of Veterinary Medicine of Serbia, Virology Department, Belgrade, Serbia to the EURL for CSF (Hannover, Germany) to confirm the CSF outbreak. After CSF diagnosis remaining sample material was used in the present study.

### Cells

Three different cell lines [Porcine kidney (PK15), PK15(A) [24], and swine testis epitheloid (STE)] were used for virus isolation. The cell lines PK15 (CCLV0051) and STE (CCLV0255) were obtained from the Collection of Cell Lines in Veterinary Medicine (CCLV, Friedrich-Loeffler-Institute, Island of Riems, Greifswald, Germany). These cell lines are routinely used for virus isolation at the EURL for CSF. Transfection experiments were performed using swine kidney (SK6) cells according to a previously established protocol [17]. SK6 cells were kindly provided by the Institute of Virology and Immunoprophylaxis, Mittelhäusern, Switzerland [25]. PK15(A), PK15 and STE cells were cultivated in Eagle´s Minimum Essential Medium (EMEM) supplemented with 5% [PK15(A)] or 10% fetal calf serum (PK15 and STE) which had been tested for absence of pestivirus genomes and pestivirus-specific antibodies. After infection the cells were maintained in EMEM supplemented with 10% fetal calf serum. SK6 cells were grown in Dulbecco´s modified Eagle´s medium supplemented with 10% fetal calf serum.

### Isolation of viral RNA, real-time RT-PCR, and nucleotide sequencing

Viral RNA was extracted from organ samples using QIAzol (Qiagen, Germany) combined with the RNeasy Mini Kit (Qiagen, Germany). RNA preparation from leucocytes and swab samples was performed with the QIAamp RNA Blood Mini Kit and the QIAamp Viral RNA Mini Kit (Qiagen, Germany) according to the manufacturers’ recommendations. The concentration of isolated RNA was determined photometrically (Nanodrop, Thermo Scientific). Viral RNA was detected by a CSFV-specific quantitative reverse transcriptase polymerase chain reaction (qRT-PCR) as described previously [26]. *In vitro* run-off transcripts of target RNA were used as copy standard for quantification of CSFV-specific RNA copies per μg RNA. Afterwards the genome equivalents (RNA copies per electroporation reaction) were calculated on the basis of the RNA amount used for electroporation (Table 1). For sequence analysis, the genomic region encoding the structural protein E2 was amplified by conventional RT-PCR and subjected to nucleotide sequencing as described previously [9].

**Table 1:** Conventional virus isolation and virus recovery after RNA electroporation testing organ samples from experimentally infected pigs.

CSFV strain (catalogue number ^a^)		Virus isolation			qRT-PCR		RNA transfection of SK6 cells		
	Sample [animal ID-organ-days post infection]	PK15	PK15(A)	STE	C_q_ value	copies /μg total RNA	Amount of total RNA [μg/reaction]	Genome equivalents [copies/reaction]	Recovery of infectious virus^b^
Rösrath (CSF1045)	261-spleen-26	+	+	+	23.05	4.2 x 10^4^	8.8	3.7 x 10^5^	1:4,000
	261-kidney-26	+	+	+	24.07	1.9 x 10^4^	10.5	1.9 x 10^5^	1:40
	261-tonsil-26	+	+	+	20.76	1.5 x 10^5^	17.6	2.7 x 10^6^	1:400
	261-lymph node-26	+	+	+	20.27	1.1 x 10^5^	32.9	3.7 x 10^6^	1:4,000
Brescia (CSF0947)	283-spleen-09	+	+	+	21.62	5.8 x 10^5^	2.6	1.5 x 10^6^	1:400
	283-kidney-09	+	+	+	27.10	2.9 x 10^3^	8.1	2.4 x 10^4^	-
	283-tonsil-09	+	+	+	26.68	3.4 x 10^4^	0.9	3.0 x 10^4^	1:4
	283-lymph node-09	+	+	+	25.53	2.3 x 10^4^	3.0	6.9 x 10^4^	-
	284-spleen-07	+	+	+	27.23	9.2 x 10^3^	2.4	2.2 x 10^4^	1:40
	284-kidney-07	-	-	-	28.61	1.8 x 10^3^	4.7	8.5 x 10^3^	1:4
	284-tonsil-07	+	+	-	28.54	4.9 x 10^3^	1.8	8.9 x 10^3^	-
	284-lymph node-07	-	+	+	30.08	4.8 x 10^2^	6.5	3.1 x 10^3^	-
Koslov	285-spleen-09	-	-	+	26.13	7.1 x 10^3^	9.8	7.0 x 10^4^	1:4
	285-kidney-09	-	-	-	32.30	1.9 x 10^2^	3.6	6.9 x 10^2^	-
	285-tonsil-09	-	-	+	27.86	8.8 x 10^3^	2.4	2.1 x 10^4^	1:4
	285-lymph node-09	-	-	+	29.79	4.6 x 10^2^	12.5	5.7 x 10^3^	1:4

^a^ = Catalogue numbers deposited in the virus database of the EU Reference Laboratory for Classical Swine Fever Virus;

^b^ = Dilution of transfected cells with a positive result for virus recovery;

+ = virus isolation from sample material was positive;

- = virus isolation from sample material was not possible

### Virus isolation

Virus isolation was performed according to the standard protocols [4, 5]. Briefly, organ samples were homogenized and two concentrations of the organ suspension (undiluted and 1:10 diluted) were incubated on the cells. After two passages cell culture supernatant was collected and the cells were fixed by heat treatment (80°C for 3 h). For detection of viral antigen an indirect immune-peroxidase staining was performed using an NS3-specific monoclonal mouse antibody BVD/C16 ([27], dilution 1:25 in PBS-0.01%Tween) and a polyclonal rabbit anti-mouse horseradish peroxidase conjugate (dilution 1:200 in PBS-0.01% Tween, Catalogue no.: P0260, DAKO, Denmark).

### Transfection of RNA

Electroporation of SK6 cells was performed as previously described with slight modifications [17]. Briefly, confluent SK6 cells, which were grown in a 10 cm dish, were resuspended in 0.4 ml PBS without Ca^2+^ and Mg^2+^. For transfection, SK6 cells were mixed with RNA and added to a 0.2 cm cuvette before the pulse [950 μF and 180 V; Gene Pulser II (Bio-Rad, Germany)]. For the first set of samples different amounts of total RNA were used for transfection, ranging from approximately 1 μg to 33 μg of total RNA (corresponding to 10–15 μl; Table 1). Transfected cells were counted and diluted in 10-fold serial dilutions starting with a 1:4 dilution. They were seeded together with 1.5–2.0 x 10^6^ naïve cells in six-well dishes. For RNA prepared from field samples a constant amount of 1 μg was used for electroporation (Table 2). Three days post transfection cells were fixed by heat treatment (80°C for 3 h). CSFV replication was detected by an indirect immunofluorescence assay using the NS3-specific monoclonal mouse antibody BVD/C16 (dilution 1:25 in PBS-0.01%Tween; incubation at 37°C for 2 h), and a goat anti-mouse IgG-Cy3 (Catalogue no.: 115-165-003, Dianova, Germany, dilution 1:800 in PBS-0.02% Tween; incubation at 37°C for 1 h). After each incubation step the cells were washed three times with PBS-0.05% Tween. Finally the wells were filled with ultrapure water and immunofluorescence was analyzed by an immunofluorescence microscope (Leica DFC420C, Germany).

**Table 2:** Conventional virus isolation and virus recovery after RNA electroporation testing field samples.

Sample ID	Virus isolation			qRT-PCR		RNA transfection of SK6 cells	
	PK15 cells	PK15(A) cells	STE cells	C_q_ value	copies /μg total RNA	Genome equivalents [copies/reaction]	Recovery of infectious virus
154–1	+	+	+	24.35	3.2 x 10^4^	3.2 x 10^4^	+
154–2	+	-	+	22.56	9.3 x 10^4^	9.3 x 10^4^	+
154–3	+	-	+	21.45	5.0 x 10^5^	5.0 x 10^5^	+
154–4	+	-	-	21.35	1.5 x 10^6^	1.5 x 10^6^	+
154–5	+	+	-	27.90	1.5 x 10^4^	1.5 x 10^4^	+
154–6	-	-	-	28.43	1.5 x 10^3^	1.5 x 10^3^	+
154–7	-	-	+	27.38	1.2 x 10^4^	1.2 x 10^4^	+
154–8	+	+	+	20.83	4.9 x 10^5^	4.9 x 10^5^	+

+ = virus isolation from sample material was positive;

- = virus isolation from sample material was not possible

### Ethics statement

The animal study followed the animal welfare regulations and standards according to EU Directive 201/63/EU and institutional guidelines. It was approved by the Specialized Department of Animal Welfare Service of the LAVES (Permit Number: LAVES AZ 11/0565) including recommendations of the ethics committee which was appointed by the LAVES according to the German animal welfare act (Tierschutzgesetz, § 15, Abs. 1, Nr. 2). Herewith we confirm that this ethic committee is the competent ethics authority and that no other specific Institutional Animal Care and Use Committee for the performance of the animal experiment were required. On the basis of a CSF clinical signs scoring system [28] the general health of study animals was recorded at least twice daily from three days before inoculation until completion of the study by experienced animal husbandry personnel and veterinarians. If the animals were in moribund stage they were euthanized by an intravenous overdose of pentobarbital followed by exsanguination (heart puncture) to minimize suffering. Since the clinical picture of animals infected with the CSFV strain Koslov is described in several publications, the number of animals of the control group was reduced. In addition, sample material (e.g. serum and organ samples) of the animals was used for teaching purposes and stored in the EURL reference material archive for further use in international ring trials.

### Experimental design of the animal study

The animal experiment was carried out at the high containment unit of the EURL (Hannover, Germany). Eight pigs (37–47 days old; 12–15 kg) of both genders were purchased from a breeding farm of the University of Veterinary Medicine Hannover, Germany. They were tested negative for pestivirus genomes and for neutralizing antibodies against pestiviruses and were randomly allocated into two groups comprising five (RNA inoculation group, animal no: 355 to 359) and three animals (infection control group, animal no: 388 to 390), respectively. The animals were housed in two separate fenced pens under high containment conditions with no direct and indirect contact. They were kept litter-less, were fed with commercial pig feed and had access to water *ad libitum*. Animals of the RNA inoculation group were oronasally inoculated with 2 ml EMEM containing 3.3 μg RNA which was prepared from whole blood of an animal infected with highly virulent CSFV strain Koslov. RNA transfection of SK6 cells and subsequent serial dilution of the transfected cells was used to calculate the infection equivalents and to confirm that the RNA was infectious after transfection of susceptible cells. The infection equivalents were determined on the basis of the highest dilution of transfected cells with a positive result for virus recovery and the RNA amount used for inoculation of one animal. Accordingly, each animal was inoculated with ~1.3 x 10^3^ infection equivalents. Additionally, to show that material used for RNA preparation is *per se* infectious, three weaner pigs were oronasally inoculated with the original material (control group). The inoculation volume was equal to the volume used for RNA preparation and corresponds to the infection dose of 1.13 x 10^5^ TCID_50_/ animal.

Rectal temperatures and clinical signs (according to Mittelholzer et al. 2000 with slight modifications, [28]) of all weaner pigs were recorded from three days before inoculation until euthanasia of the animals. Slight modifications were made for scoring parameters 9 “Defecation” and 10 “Leftovers in feeding trough”. The criterion mild diarrhea was added to parameter 9 “Defecation” and scored with one point. Parameter 10 “Leftovers in feeding trough” could not be recorded for each animal because the animal groups were housed in one pen with one trough. Thus, the possible total number of scoring points per animal was lower than for the scoring scheme according to Mittelholzer et al., 2000. On day 1, 2, 3, 5, 7, 10, 14, 17 and 21 post inoculation blood samples (full blood and anti-coagulated EDTA blood) as well as nasal and pharyngeal swabs were taken and subjected to virological (qRT-PCR) and serological testing (virus neutralization assay). Leucocyte and thrombocyte counts were determined using a hematology analyzer (Abacus Junior vet/130464, Guder Medizintechnik, Germany). On the day of euthanasia, each animal was subjected to post-mortem examination. Organ samples (tonsil, lymph nodes, spleen, and kidney) were collected and subjected to virological testing (qRT-PCR). The ARRIVE (Animal Research: Reporting of *In Vivo* Experiments) Guidelines Checklist of this animal experiment is available as supporting information (S1 File).

### Virus neutralization assay

Virus neutralization assay was performed with serum samples that were taken at seven days post inoculation and later on. The assay was performed according to the standard protocols [4, 5].

## Results

### Virus rescue from experimentally derived organ samples

Sixteen organ samples from four experimentally infected animals were analyzed by a CSFV-specific qRT-PCR to quantify the viral genome load of each individual sample. All samples were tested positive for CSFV genome. The CSFV genome loads ranged between 1.9 x 10^2^ and 5.8 x 10^5^ genome equivalents/ μg RNA [quantification cycle (C_q_) values from 20 to 30] (Table 1). Conventional virus isolation using PK15 cells was successful from ten out of 16 samples. The use of two additional cell lines resulted in virus isolation from 11 out of 16 [PK15(A) cells] and from 13 out of 16 (STE cells) samples, respectively. With regard to the four samples taken from Koslov infected pigs, virus isolation was more efficient on STE cells than on PK15 and PK15(A) cells. In routine CSFV diagnosis differences in efficiency of virus isolation on various CSFV susceptible cell lines are known. To our knowledge, those differences have not been systematically analyzed. Therefore, more than one cell line is usually used for virus isolation. For two samples (284-kidney and 285-kidney) virus isolation was not possible. Genome loads of these samples were 1.8 x 10^3^ (284-kidney) and 1.9 x 10^2^ (285-kidney) genome equivalents/ μg RNA, respectively (Table 1). To compare virus isolation with virus recovery after RNA transfection, total RNA from the same organ material previously subjected to qRT-PCR analysis (see above) was transfected into SK6 cells by electroporation. Emergence of virus was observed for 11 out of 16 samples. Remarkably, for sample 284-kidney recovery of infectious virus was possible only after RNA transfection whereas this sample was tested negative in conventional virus isolation on three different susceptible cell lines (Table 1). In general, the transfection of more than 1.9 x 10^5^ genome equivalents was highly efficient. In this case, most of the samples were still positive after diluting the transfected cells 1:400 or 1:4,000 in suspensions of naïve cells. For six samples even transfection of total cellular RNA containing 5.7 x 10^3^ to 7.0 x 10^4^ genome equivalents allowed virus recovery. In summary, virus rescue by RNA transfection was successful from each of the four experimentally infected pigs (Table 1).

Despite the use of antibiotics, microbial contamination was a problem for inoculation of homogenized organ samples on cells. For three out of 16 organ samples bacterial contamination of cells was observed during first passage of virus isolation. After transfection of SK6 cells with RNA isolated from the same samples CSFV was recovered without facing any problems with microbial contamination.

### Virus rescue from field samples

To test whether RNA transfection can also be used as a general diagnostic tool, eight organ suspension samples taken during a CSFV outbreak in Serbia in 2010 were tested. Applying conventional virus isolation, six out of eight samples were tested positive on PK15 cells. Inoculation of these samples on two additional cell lines resulted in virus recovery from three [PK15(A) cells] and five (STE cells) samples, respectively. The CSFV genome copy numbers of these samples ranged between 1.5 x 10^3^ and 1.5 x 10^6^ copies/ μg RNA (C_q_ values from 21 to 28) (Table 2). Since it was shown that electroporation using ~1 μg total RNA was efficient, SK6 cells were transfected with 1 μg total RNA. Virus recovery was successful for all samples using one single cell line for transfection (Table 2). In comparison to this, three different cell lines were needed to rescue infectious virus by inoculation of organ material on susceptible cells for most of the samples. As already described for the experimentally derived organ samples, microbial contamination was also observed after inoculation of cells with suspension of field samples. For three out of eight organ samples at least one virus isolation set-up (concentrated organ homogenate or 1:10 dilution of organ homogenate) was contaminated during first virus passage. After transfection of SK6 cells with isolated RNA none of the cell cultures were affected by bacterial contamination. Furthermore, E2 coding sequences of two rescued viruses (sample no. 154-1 and 154-8) were 100% identical to the respective sequences obtained from the RNA of the original sample material.

### Oronasal inoculation of pigs with RNA from infectious sample material

**Fig 1:**
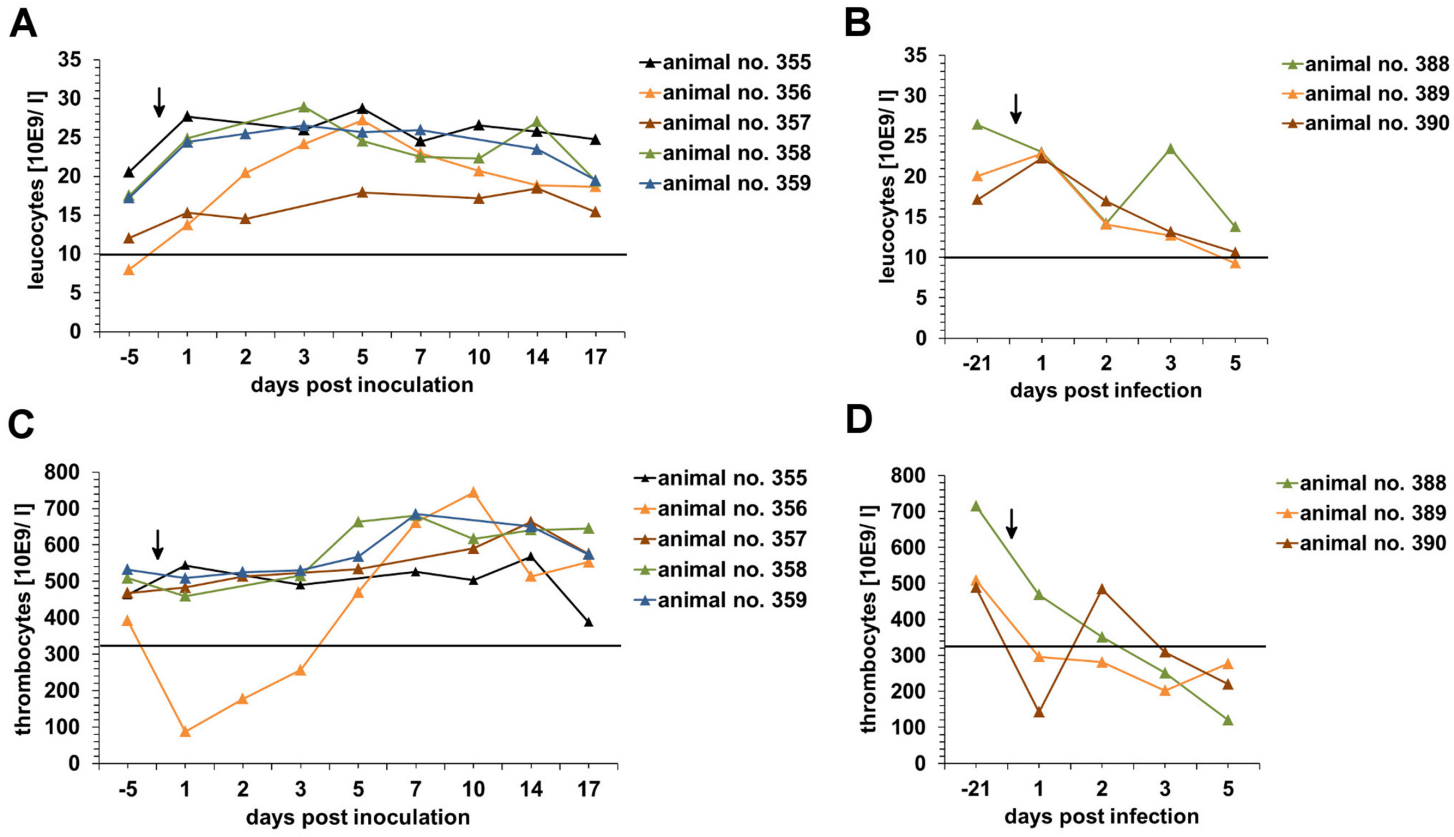
Leucocyte and thrombocyte counts of animals inoculated with RNA or infected with CSFV Koslov. Anti-coagulated EDTA blood samples were taken 21 (control group) or 5 days (RNA inoculation group) before inoculation of the animals and on day 2, 3, 5, 7, 10, 14 and 17 after inoculation. Inoculation or infection of the animals is marked by an arrow. Leucocyte (A and B) and thrombocyte counts (C and D) were determined using hematology analyzer. Black lines within the graphs mark the cut-off value for leucopenia or thrombocytopenia, respectively. Due to ethical reasons the animals of the control group were also part of a second study and therefore inoculated with CSFV Koslov 21 days after initial sampling.

**Fig 2:**
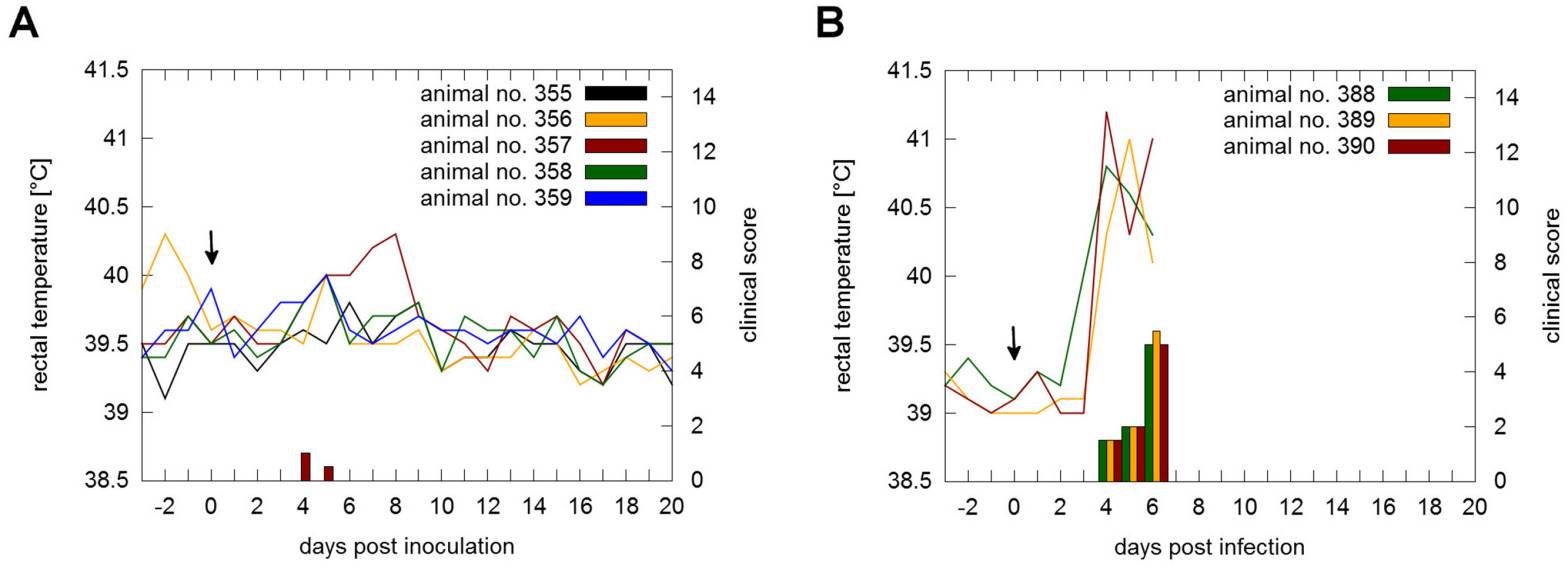
Rectal temperatures and clinical scores of animals inoculated with RNA or infected with CSFV Koslov. Temperatures and clinical signs of the RNA inoculation (A; animal no.: 355 to 359) and infection control group (B; animal no.: 388 to 390), respectively, were recorded daily starting three days prior inoculation. Inoculation or infection of the animals is marked by an arrow. Body temperatures ≥ 40.0°C for two consecutive days were considered to be fever.

To answer the question whether RNA isolated from infectious sample material, can be regarded as non-dangerous goods in case of shipment an animal experiment was performed. All five animals which were inoculated with RNA prepared from blood of an animal infected with the highly virulent strain Koslov showed no clinical signs referred to CSFV infection. A slight increase of leucocyte counts was detected after RNA inoculation which reached a plateau (~25 x 10E9/ l) on five days post inoculation (Fig 1A). No leucopenia was observed and thrombocyte counts (325 x 10E9/ l - 715 x 10E9/ l) were in physiological range except for one animal (animal no.: 356) during the first three days after inoculation (Fig 1C). Analysis of serum samples taken on day 7, 10, 14, 17 and 21 post inoculation by virus neutralization assay demonstrated the absence of CSFV-specific neutralizing antibodies. Furthermore, swabs, leucocytes and organ samples (lymph node, tonsil, spleen and kidney) were tested negative for CSFV genome. One animal (animal no.: 357) showed a slightly increased body temperature (40°C) between five and eight days post inoculation. This animal showed mild diarrhea for two days on four (clinical score = 1) and five (clinical score = 0.5) days post inoculation (Fig 2A). However, samples taken from this animal were all tested negative for CSFV genome or antibodies.

The RNA used for animal inoculation was only infectious after transfection of SK6 cells. Direct inoculation of SK6 cells with RNA mixed in culture medium lead to no virus production whereas virus was detected for an included positive control (inoculation of the cells with infectious cell culture supernatant containing CSFV Alfort/187).

In contrast to the RNA inoculation group, animals of the infection group developed high fever on day three post infection, showed severe clinical signs of CSF, and were finally euthanized on day seven post infection in a moribund stage (Fig 2B). A decrease of leucocyte and thrombocyte counts was observed after infection (Fig 1B and 1D). Since the animals were euthanized at an early time point after infection, the presence of virus neutralizing antibodies was not expected and therefore not determined. In contrast to the RNA inoculation group, low CSFV genome loads were already detectable in leucocytes taken on day two post inoculation (C_q_ values 34.98 to 37.41), followed by an increase until last sampling day (C_q_ values 21.08 to 25.82). Swabs were tested positive on day three post infection (C_q_ values 38.17 to 38.3) until last sampling day (C_q_ values 29.17 to 32.76). Furthermore, high copy numbers of CSFV RNA were detected analyzing the organ samples of the control group (C_q_ values 20.9 to 31.29).

## Discussion

Virological methods for the diagnosis of CSF are based on virus isolation after incubation of sample material on susceptible cells and detection of virus antigen and virus genome [4]. RNA transfection methods may represent a good alternative to rescue positive stranded RNA viruses from samples that are negative in virus isolation [20–23]. So far, knowledge on efficiency of virus rescue by RNA transfection directly compared to conventional virus isolation using samples of CSFV infected pigs is still missing. To conclude whether RNA transfection can be recommended in addition to conventional virus isolation, the present study focused on direct comparison of both methods using organ samples of experimentally and field infected pigs, respectively. It was demonstrated that conventional virus isolation was time and labor intensive as for several samples more than one cell line had to be used for successful virus isolation. RNA transfection was successful in particular for critical samples that were only positive for one out of three tested cell lines. For two samples (284-kidney, 154–6), rescue of infectious virus was possible only after RNA transfection but not with the conventional virus isolation, even when three different cell lines were inoculated. However, virus rescue by RNA transfection showed also limitations which might be due to the lack of integrity of the 12.3 kb CSFV RNA genome after RNA preparation. In previous studies similar observations have been reported [20, 21, 23]. Since determination of CSFV genome copy numbers is based on amplification of a short genome region, this method cannot provide information on the actual number of infectious viral genomes. Nevertheless, it can be assumed that the amount of intact CSFV genomes increases when high amounts of genome equivalents are used for transfection. Furthermore, efficiency of RNA transfection for recovery of virus is dependent on several other factors, including amount of RNA, sample quality, and cell viability after transfection. The results of our study showed, that virus rescue after transfection of 1.9 x 10^5^ CSFV genome equivalents was very efficient. For these samples, infectious virus was recovered even in high dilutions of transfected cells. Genome equivalents in this range can be expected in 10 to 15 μl volumes of RNA preparations with C_q_ values between 20 and 24 in qRT-PCR. However, it was also shown that samples with higher C_q_ values (25 to 30) allow virus rescue by RNA transfection. The minimal amount of extracted RNA resulting in successful virus rescue was tested using field samples from an outbreak in Serbia. One microgram of total RNA, which contained between 1.5 x 10^3^ and 1.5 x 10^6^ CSFV genome equivalents, was sufficient for successful virus rescue of all field samples. From the methodological point of view, RNA transfection is less labor and time intensive and not prone to microbial contamination of cell culture. It is well known that microbial contamination can significantly limit the efficiency of virus isolation after inoculation of cells with infectious sample suspension. In this context, it seems to be likely that bacterial contamination observed for sample 284-kidney was at least partly responsible for the failure of conventional virus isolation although virus rescue by RNA transfection was successful. Furthermore, it is known that autolyzed sample material has cytotoxic effects on cell culture as well [4]. Insufficient quality is often a problem of diagnostic samples obtained from wild boar and in such cases RNA transfection might be a valuable alternative. In a recently published study on FMDV it was also described that RNA transfection represents a good alternative for virus rescue when conventional virus isolation is not possible [21]. Thus it can be concluded, that RNA transfection may represent a valuable tool for generation and isolation of other positive stranded RNA viruses as well.

For a clear classification of RNA as non-hazardous material the innocuity of RNA isolated from samples of CSFV infected pigs, has to be assessed in the natural host. To our knowledge inoculation studies of pigs or wild boar via oronasal route with prepared RNA have not been reported before. The results of the present study show that experimental oronasal inoculation of RNA from the highly virulent CSFV strain Koslov did not result in infection or disease of pigs. Since CSFV Koslov causes a severe acute disease with 100% lethality, this strain has been frequently used as challenge virus and therefore is an appropriate read-out system to proof the non-hazardous character of the RNA preparations used for inoculation of pigs [29–33]. As expected, severe clinical signs were observed for the animals of the infected control group and all animals had to be euthanized in a moribund stage on day seven post infection. Absence of clinical signs, viral genomes and CSFV specific antibody response gave no hints that CSFV RNA might be *per se* infectious and hazardous for pigs after oronasal exposure. Since neither CSFV genomes nor CSFV-specific antibodies were present in any of the samples, it can be concluded that the mild unspecific clinical signs observed for animal 357 and the transient decrease of thrombocyte counts after RNA inoculation detected for animal 356 were not due to CSFV infection. In the present study pigs were inoculated with 3.3 μg of RNA corresponding to a dose of ~10^3^ TCID_50_ produced after electroporation of susceptible cells. Similar amounts of RNA material are likely to be handled and transported between laboratories. To our knowledge, the minimal infectious dose of highly virulent CSFV strain Koslov has not been determined so far. However, previous studies have shown that an inoculation dose between ~10^2.5^ and 10^3^ TCID_50_/ animal of virulent CSFV strains (Brescia and Alfort/187) resulted in animal infection and disease [34, 35] and a dose of 200 TCID_50_/ animal was used for challenge infection with CSFV Koslov [36]. Based on these facts, a dose of 10^3^ TCID_50_/ animal of the highly virulent strain Koslov can be expected to be above the minimal infectious dose. Taken into account the amount and quality of RNA used for the inoculation of pigs described in the present study together with the assumption that exposure of pigs with RNA samples represents a very rare event, it seems to be very unlikely that an accidental oral up-take of RNA sample will lead to infection of pigs.

Biosecurity classification during transportation of samples containing full length genomes of other positive stranded RNA has to be estimated for each RNA virus separately and depends also on legislation of the country. For example in the United Kingdom samples containing full length genome of FMDV require the same biosecurity level as described for infectious sample material. This includes that only shipment to laboratories with appropriate containment facilities is allowed [37].

Taken together, RNA transfection is a highly efficient approach for obtaining infectious CSFV isolates. It represents a valuable alternative to conventional virus isolation in particular for samples from which infectious virus cannot be isolated or when infectious material is not available. Accordingly, it is recommended to include the method of virus rescue by RNA transfection as an alternative approach for virus isolation in the Diagnostic Manual for CSF (Commission Decision 2002/106/EC). Furthermore, this study revealed that RNA prepared from samples of CSFV infected pigs, at least for the RNA amount used in the animal experiment described here, is *per se* not infectious for the host species and can therefore be classified as non-hazardous material. Nevertheless, the presented data highlighted that RNA can be converted relatively easy into hazardous material after transfection. Appropriate security arrangements to prevent intentional misuse or unintentional contamination (e.g. transfection reagents) are still required, although these security arrangements may not be the same as for infectious virus. The RNA transfection method described here can contribute to facilitate the exchange of virus isolates among national and international reference laboratories for CSFV as well as other authorized laboratories.

## Supporting Information

S1 File ARRIVE guidelines checklist of the RNA inoculation animal experiment. (PDF)
